# Effects of Dwell Time on the Deformation and Fatigue Behaviour of A356-T7 Cast Aluminium Alloys Used in High Specific Power IC Engine Cylinder Heads

**DOI:** 10.3390/ma13122727

**Published:** 2020-06-15

**Authors:** Elanghovan Natesan, Knut Andreas Meyer, Stefan Eriksson, Johan Ahlström, Christer Persson

**Affiliations:** 1 Department of Industrial and Materials Science, Chalmers University of Technology, 412 96 Gothenburg, Sweden; knut.andreas.meyer@chalmers.se (K.A.M.); johan.ahlstrom@chalmers.se (J.A.); christer.persson@chalmers.se (C.P.); 2 Analysis and Verification, Volvo Car Corporation, 405 31 Gothenburg, Sweden; stefan.a.eriksson@volvocars.com

**Keywords:** cylinder head, mechanical properties, dwell, fatigue, deformation behaviour, cast aluminium

## Abstract

The electrification of automotive powertrains in recent years has been driving the development of internal combustion engines towards reduced volumes with higher power outputs. These changes place extreme demands on engine materials. Engineers employ the computer-aided engineering approach to design reliable and cost-effective engines. However, this approach relies on accurate knowledge of the material deformation and fatigue characteristics during service-like loading. The present study seeks to investigate the effect of dwell times on the deformation and fatigue behaviour of the A356-T7 + 0.5 wt.% Cu alloy used to cast cylinder heads. In particular, we study the effect of dwell time duration at various temperatures. A combined fatigue-dwell testing procedure, with the dwell at the maximum compressive strain, replicates the service conditions. It is found that the material exhibits a stress relaxation behaviour with a decreasing relaxation rate. At lower temperatures, the load level influences the relaxation more than at elevated temperatures. However, the dwell does not significantly affect the hardening behaviour or the life of the tested alloy. Finally, we model the time-dependent material behaviour numerically. The Chaboche model, combined with a Cowper–Symonds power-law, is found to capture the visco-plastic deformation behaviour accurately.

## 1. Introduction

The continued adoption of electrified powertrains in recent years [1,2,3,4] has exposed the internal combustion engine materials to an increased number of start–stop thermal load cycles. When coupled with trends to increase the power of internal combustion engines while reducing their size [5], the cylinder heads in combustion engines are now subjected to higher thermal loads applied at a greater frequency than ever before. Development of reliable computational methods and life models is essential to enable computer aided engineering (CAE) life predictions of the cylinder head designs [6,7]. To shorten development times and reduce costs, greater emphasis is laid on finite element methods to virtually simulate the load conditions and predict the structural response and component lives in place of relying primarily on expensive physical tests [7]. Owing to the large low frequency plastic strains associated with the start–stop thermomechanical fatigue cycle, we often rely on strain controlled fatigue testing methods [6,7,8] to develop reliable deformation and life models. Creep or stress relaxation associated with the compressive strains that are sustained during the driving cycle of the combustion engine powertrains are well documented in theory [6,7], but need further investigation for the commonly used alloys for the manufacturing of the cylinder heads.

A cylinder head is the structure that serves to seal the combustion chamber and acts as the channel for feeding air and fuel to the engine cylinders [9,10]. In recent decades, aluminium alloys in cast form are the material of choice for mass producing components like cylinder heads that often have complex and intricate geometries. Aluminium alloys enable light-weight designs, have good thermal conductivity, and allow components to be produced with tight geometrical tolerances [9,11]. In terms of alloying aluminium, varying levels of magnesium, manganese, iron, and silicon are used to tailor the mechanical properties of the alloy. Silicon typically improves the castability of aluminium, and decreases production costs by lowering the melting temperature of aluminium, while also minimizing the solidification related contraction [12]. Addition of sodium and/or strontium is adopted to suppress the nucleation of silicon particles; a finer microstructure is obtained when the nucleation of silicon is offset to lower temperatures [12]. The high temperature strength is often improved by copper additions [13]. However, copper cannot be used in high concentrations as it has a detrimental effect on the flowability of the alloy in the cast. Consequently, it affects the high cycle fatigue strength of the material owing to increased porosity in the structure [9]. While iron has a deleterious effect on the ductility, it does enhance the mould-sticking of the molten alloy. Manganese is used to counteract the harmful effects of iron on the ductility of the alloy [10]. Older generation of cylinder head materials like A356 (AlSi7Mg0.3) offered good mechanical properties at low and medium temperatures. However, they were found to be inadequate for modern combustion engine designs owing to their reduced performance at elevated temperatures [9]. Hence, alloys with greater copper additions like A356 + 0.5 wt.% Cu are preferred for high specific power engine cylinder heads. The Mg_2_Si precipitate strengthening in the A356 family of alloys offers good creep strength at elevated temperatures [13]. Alloying additions to improve the strength and creep resistance often compromise the thermal conductivity of the aluminium alloy, so various combinations optimized for the particular designs are employed in practice [9,10].

Hold times at elevated temperatures in combination with repeated cyclic plastic deformation are of interest as they relate to the engine start-run-stop cycle [7,14,15]. While the engine start-up is associated with generating compressive plastic strains in the hot sections of the cylinder head, the continued operation of the engine results in a high temperature hold time with constant total strain leading to stress relaxation [13,14]. This “dwell” at elevated temperatures could potentially cause both mechanical damage and microstructural changes in the material, resulting in a reduced number of cycles to failure compared with a continuously loaded sample [14,16,17]. As the engine is turned off, the deformed material experiences plastic deformation again, this time in the tensile direction. When this start-run-stop cycle is repeated, fatigue cracks initiate in the structure owing to the thermo-mechanical fatigue (TMF) loading [14] affecting the durability of the cylinder head structure and reducing its useful life. It is thus of practical interest to study the effect of such dwell times at elevated temperatures on the cyclic deformation and fatigue behaviour of the alloys used in cylinder head structures to develop reliable numerical models to enable realistic computer aided engineering (CAE) efforts in engine and cylinder head design.

Relevant research of creep and dwell time effects on the number of cycles to failure of Al-Si-Mg-Cu and A356 group of cast aluminium alloys is briefly reviewed here. Azadi et al. [18] studied the effect of dwell times with compressive mechanical strains at elevated temperatures using out of phase thermo-mechanical tests. They concluded that the dwell time had no influence on the number of cycles to failure of the A356 aluminium alloy. Beck et al. [19] investigated the effect of hold time on the life of AlSi6Cu4 alloy with superimposed high cycle fatigue (HCF) loads on thermo-mechanical fatigue (TMF) load cycles. They observed that the hold time at elevated temperatures with compressive strains on its own did not affect the life of the material. However, the stress relaxation associated with the dwell, when coupled with the superimposed HCF loading associated with the combustion cycle, often resulted in tensile stresses that tend to have a life reducing effect.

Most of the dwell fatigue tests reported are carried out with the hold time at tensile loading, as that is when the dwell time influenced damage is expected in general [14]. Even so, studies by Wells et al. [20] have shown that, depending on the material, a compressive dwell could reduce the life of the component as well. Moverare et al. [16] also showed that the length of the hold time significantly influences the crack growth rate in the material during the fatigue load cycles with dwell times when studying the hold time effects on Inconel 718 group of alloys. So, it becomes imperative to study the effect of different dwell times on added damage to the material when compared with continuously loaded samples. While numerous studies have explored the effect of such dwell time on the number of cycles to failure of various alloys used in powerplants, the hot parts of jet engines and numerous high temperature application areas, there is a dearth of relevant information studying the effect of such dwell times on the high temperature fatigue behaviour of A356-T7 + 0.5 wt.% Cu cast aluminium alloys that are commonly used in modern day cylinder heads.

The durability of the material could be dependent on the number of loading cycles at low temperatures or be heavily influenced by time-dependent damage processes at elevated temperatures [21], thus it is imperative to study the effect of dwell times on the fatigue behaviour of the alloy in the temperature spanning the operating temperatures experienced by the cylinder head material. To this end, a combined fatigue-dwell test plan was implemented with the dwell times executed in between fatigue cycles to study the influence on the life of the material. The strain-controlled fatigue cycles were run at various temperatures spanning from room temperature to 250 °C and at load levels corresponding to 0.2%, 0.3%, and 0.4% total strain amplitudes with the dwell time set-up at the maximum compressive strain for different durations. A further goal of this study is to develop temperature dependent numerical models that could be used in a finite element setting to predict the visco-plastic load-response behaviour of the alloy. The series of combined fatigue and stress relaxation tests that were carried out at varying temperatures and load levels of interest are used to calibrate the Chaboche and Cowper–Symonds visco-plastic material model, which could be used to model the visco-plastic deformation behaviour of the alloy system in a CAE setting for cylinder head development.

The samples for testing are carefully extracted from the fire deck regions of cylinder heads from Volvo Cars’ (Gothenburg, Sweden) inline 4-cylinder VEP4 petrol engines. This region is particularly sensitive to thermo-mechanical fatigue [6,22]. A cylinder head often has a complex cast microstructure owing to the differing cooling rates and the section thickness, often resulting in material microstructure that cannot reliably be replicated by specially casting the test specimens. This study comes with the limitation that the presented results of stress relaxation, deformation, and fatigue behaviour of the alloy are only for an optimized, constant set of processing parameters, and consequent microstructural. The manufacturing processes have a significant influence on the nature of the castings and the resulting microstructure, even for identical chemical compositions, and can potentially have a dramatic effect on both the deformation and fatigue lives of the tested alloy.

## 2. Materials and Methods

### 2.1. Material

The measured chemical composition of the A356.0-T7 + 0.5 wt.% Cu alloy used to cast the cylinder heads is summarized in Table 1. The nomenclature of the alloy can be broken down as follows: ’A’ indicates high purity, the decimal ′.0′ indicates a cast form, the 300 series refers to Al-Si alloy systems with magnesium and/or copper additions, and T7 indicates the heat treatment applied [10].

**Figure 1:**
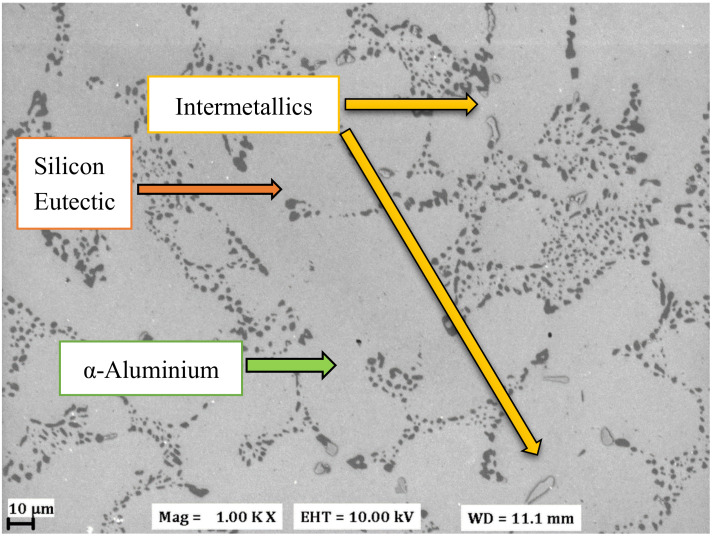
Electron micrograph of the tested cast A356-T7 + 0.5 wt.% Cu alloy.

**Figure 2:**
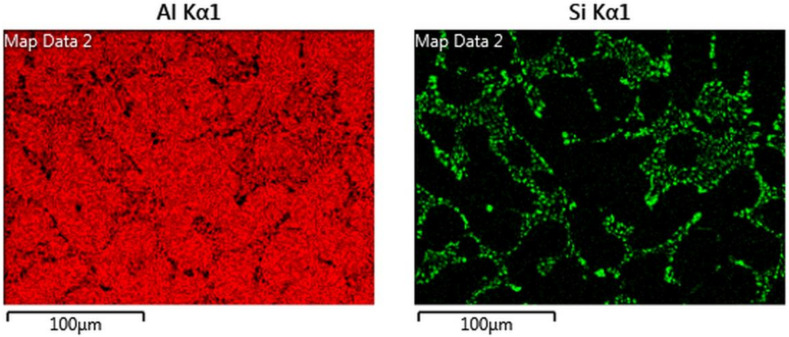
An energy dispersive X-ray spectrometer (EDS) map showing the distribution of aluminium and silicon in the tested A356-T7 + 0.5 wt.% Cu alloy.

A microstructural investigation was carried out using a scanning electron microscope (SEM) and the microscopic image obtained from a Zeiss LEO 1550 high resolution SEM (Zeiss, Jena, Germany) is presented in Figure 1. To reduce the influence of the underlying matrix material, a Gemini field emission gun (Zeiss, Jena, Germany) was used with an acceleration voltage of 10 kV. The software Aztec 3.1 (Oxford Instruments, Abingdon, England) was used to process the electron micrograph. Qualitative micro-chemical analysis was carried out with Oxford Instruments’ (Oxford Instruments, Abingdon, England) solid-state dispersive X-ray spectrometer (EDS) and the EDS maps for aluminium and silicon are presented in Figure 2. Various iron, magnesium, and copper-based intermetallics were also detected in the material microstructure using local EDS chemical analysis. The average secondary dendrite arm spacing (SDAS) was measured at 30–32 μm in the sample extraction zone using the mean linear intercept method on aligned sets of secondary cells in the microstructure.

**Figure 3:**
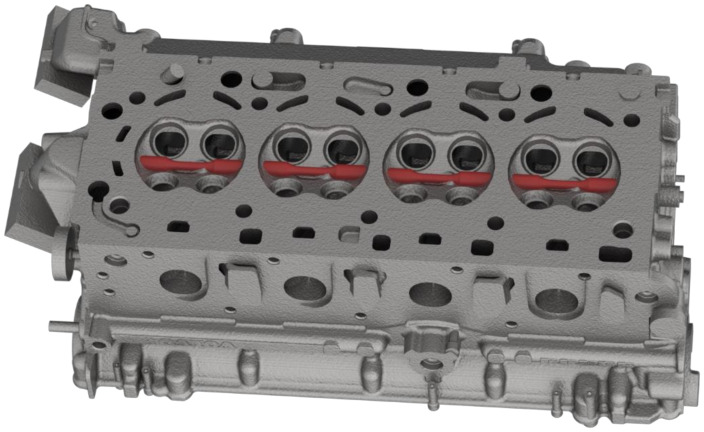
Sample extraction for testing from the valve bridge areas of the cylinder heads.

**Figure 4:**
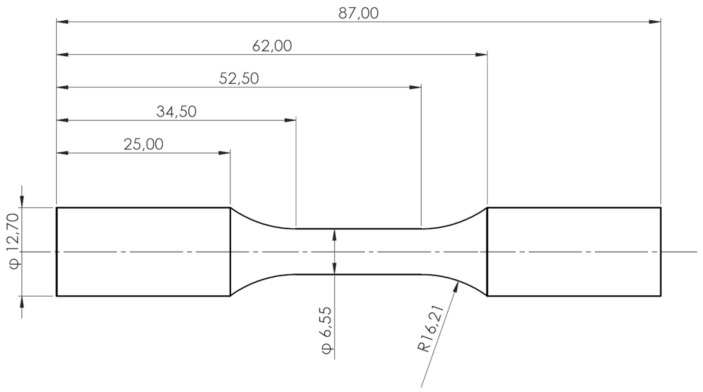
Geometry of the test bars prepared in agreement with ASTM standards [All dimensions are in mm].

The material for testing is extracted from the fire deck area of Volvo Cars’ VEP-4 inline 4-cylinder engine cylinder heads and close to the valve bridge areas, as shown in Figure 3, that are often the most sensitive to thermo-mechanical fatigue failures [10]. A description of the production process and the material microstructure is detailed in our previous research work [23,24]. The test specimens for the study were machined from the extracted material to the geometry as shown in Figure 4, keeping to the ASTM recommendations for strain controlled fatigue testing [25].

### 2.2. Testing

#### 2.2.1. Test Plan and Sample Preparation

**Figure 5:**
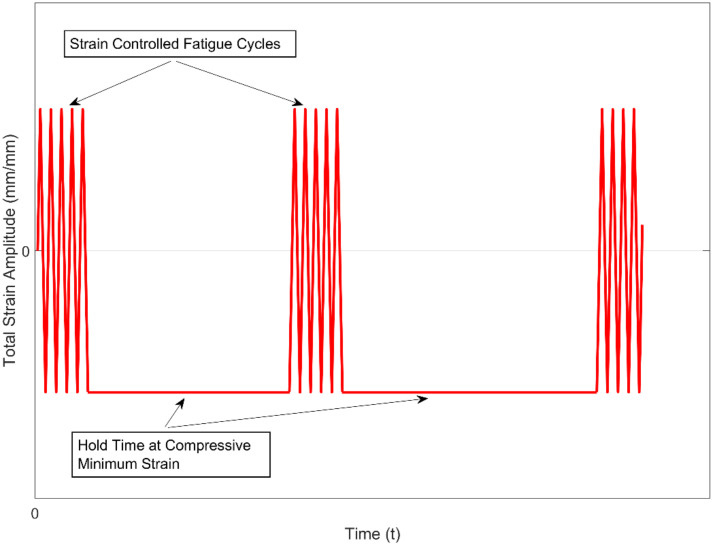
Test schematic for the combined fatigue + hold time tests.

The tests for the study were designed to capture the hold time effects on the deformation behaviour and the number of cycles to failure of the material at various isothermal temperatures. The combined strain-controlled fatigue-dwell time tests were performed as the schematic illustrates in Figure 5. The tests in this study were conducted in the temperature range spanning from room temperature to the maximum expected temperatures in the cylinder head [10]. The temperatures were selected as room temperature (RT), 150, 200, and 250 °C to enable a reliable development of numerical models that could be interpolated with reasonable accuracy in the tested range. Two series of tests were run, one with a hold time of 600 s and the other with a hold time of 3600 s, both having the hold times at the maximum compressive strain to mimic the load conditions expected in the cylinder head [26]. The tests with the 600 s hold time were run at 150, 200, and 250 °C, at three different total strain amplitudes of 0.2%, 0.3%, and 0.4% and with R_ε_ = −1. The tests with the longer hold times of 3600 s were run at 0.4% total strain amplitude and with a strain ratio R_ε_ = −1 at room temperature (RT), 150, 200, and 250 °C. The number of strain cycles before each of the dwell times is taken to be a fraction (10%) of the number of cycles to failure after uninterrupted fatigue cycling of the same material [23,24]. All of the samples were polished sequentially up to a mirror surface finish before testing. The test summary is presented in Table 2.

* Note that the terms ‘hold’ times and ‘dwell’ times are used interchangeably throughout the document, but in essence mean the same test condition of holding the control variable (here, total strain) constant for the specified amount of time.

#### 2.2.2. Test Equipment

The combined fatigue-relaxation tests were carried out using an Instron 8501 servo-hydraulic uniaxial testing machine (Instron, Norwood, MA, USA). An Instron 3119-407 test chamber (Instron, Norwood, MA, USA), which employs a forced air convection method, was used to establish the test temperatures. The measurements were sampled using a data acquisition system capable of sampling up to 1 kHz. The tests were carried out at a range of temperatures from room temperature (RT) up to 250 °C and two different extensometers were used to measure and control the said tests, and are detailed in Table 3. The material was deformed with a strain rate of 1%.s^−1^ during the strain-controlled load cycling with a triangular load wave application.

Before the loads were applied to the specimens, the sample and the test equipment were heated to the target temperature and held at isothermal conditions for 3 h. The rate at which the target temperature is reached is different for the different components of the test setup; the air in the temperature chamber is the fastest to reach the stable target temperature within a few minutes followed by the specimen. The components with higher mass and inertia, namely the grip setup and the pull rods, on the other hand, take over an hour to reach the target temperature and eventual thermal stabilization. This heating up procedure is followed rigorously to eliminate the interference of the thermal expansion of the equipment on the experimental measurements.

## 3. Results

Stress relaxation, while physically the same phenomenon as creep, is observed in structures that are held at a constant deformed shape instead of a constant load at a relatively high temperature for the material [8]. The stress relaxation is always directed towards stress reduction when strain is held constant. As with creep, the creep/relaxation rate decreases initially followed by a constant creep/relaxation rate with continued dwell.

### 3.1. Effect of Hold Time on Stress Evolution

**Figure 6:**
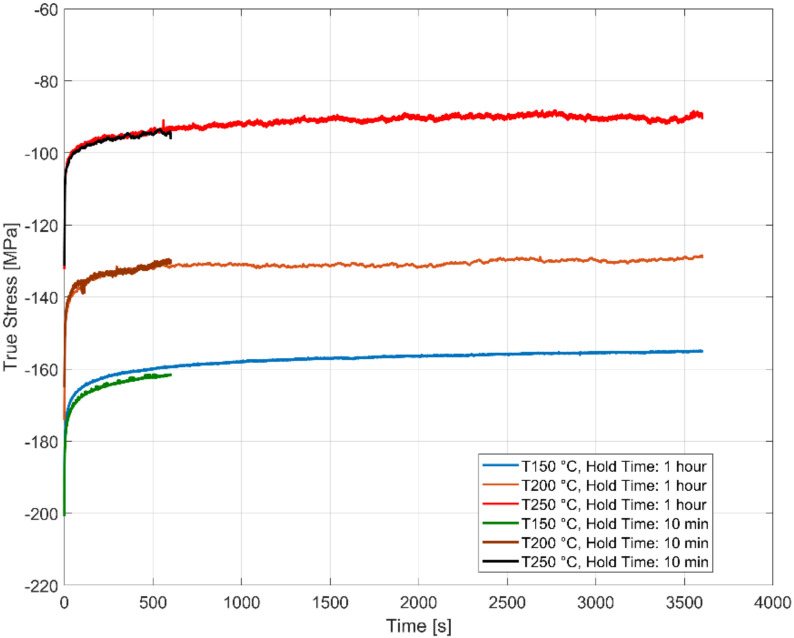
Effect of hold time on stress relaxation at various temperatures with the total strain held constant at ε_Hold_ = −0.4%.

The loss in stress with dwell time of 600 and 3600 s at a compressive total strain of 0.4% at various temperatures is presented in Figure 6. The time taken to achieve a stable stress relaxation rate follows an exponential pattern at all temperatures with a decreasing stress relaxation rate initially, and a constant stress relaxation rate is achieved rapidly thereafter in the initial few minutes of the test at all temperatures until the strain was changed.

### 3.2. Effect of Strain Amplitude during Hold Time on Stress Relaxation

**Figure 7:**
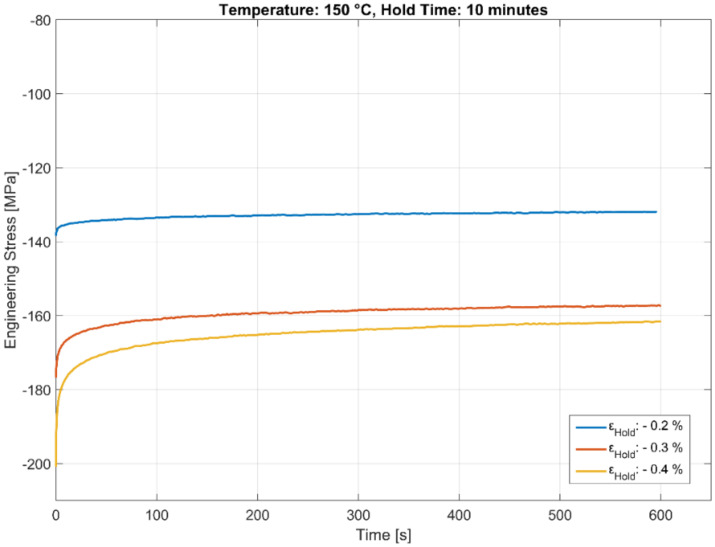
Effect of strain amplitude on stress relaxation at 150 °C.

**Figure 8:**
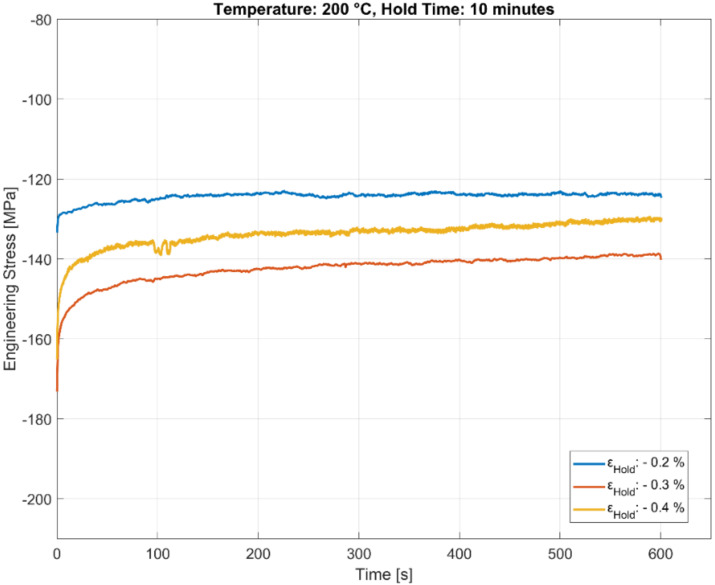
Effect of strain amplitude on stress relaxation at 200 °C.

**Figure 9:**
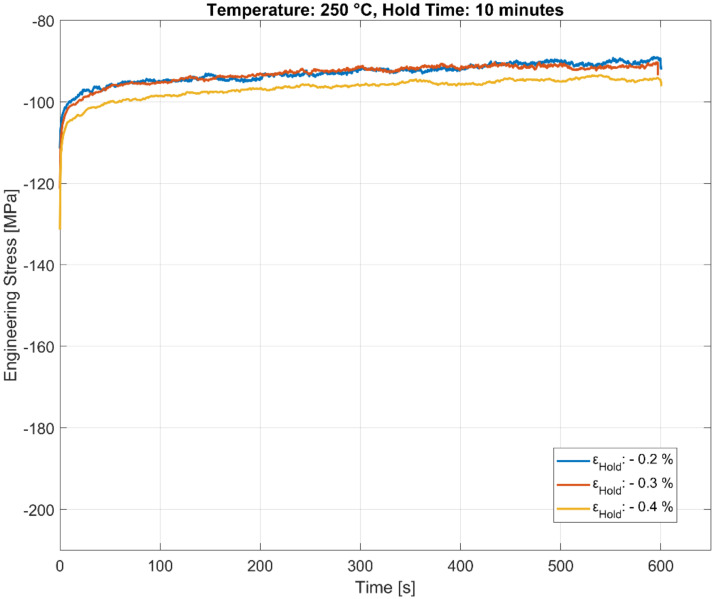
Effect of strain amplitude on stress relaxation at 250 °C.

The stress relaxation behaviour of the A356-T7 + 0.5 wt.% Cu alloy held at three different compressive total strains of −0.2%, −0.3%, and −0.4% for a hold time 600 s is presented in Figure 7, Figure 8 and Figure 9. The stable stress relaxation rate achieved after the initial stress loss when the material is held at constant strain seems to be affected by the temperature and the hold strain level, as shown in the plots. Consequently, the initial stress response during the rapid loading to the constant hold strain level will hide part of the relaxation. The strain amplitudes have a greater influence at lower temperatures than at the high temperatures owing to the varying degrees of hardening with plasticity at different temperatures. The influence of temperature could be twofold:

1. With the material exhibiting insignificant hardening with increasing plasticity at elevated temperatures, the stress relaxation measured is identical for all the tested load levels at 250 °C, in contrast to the lower temperatures, where the material exhibits considerable hardening with increasing plasticity.
2. As mentioned above, part of the explanation could be that the relaxation at the highest temperature (250 °C) is rapid enough to complete already during the compressive straining.

The scatter in the measured deformation properties for the tested A356-T7 + 0.5 wt.% Cu cast aluminium alloy is further evident when observing the initial stress response and the subsequent stress relaxation for the tests at 200 °C, where the stress response observed is more severe for the compressive loading at 0.3% than at 0.4% before the subsequent dwell, as presented in Figure 8.

### 3.3. Stress Relaxation during Multiple Hold Time Events

**Figure 10:**
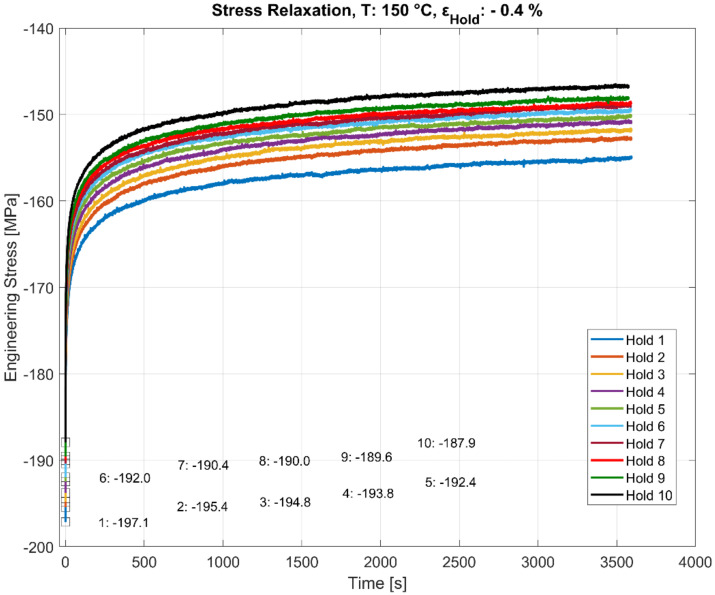
Effect of hold time on stress relaxation at 150 °C with alternating fatigue loading and hold period. The start (trough) values at the beginning of each hold period (numbered 1–10) are stated in MPa.

**Figure 11:**
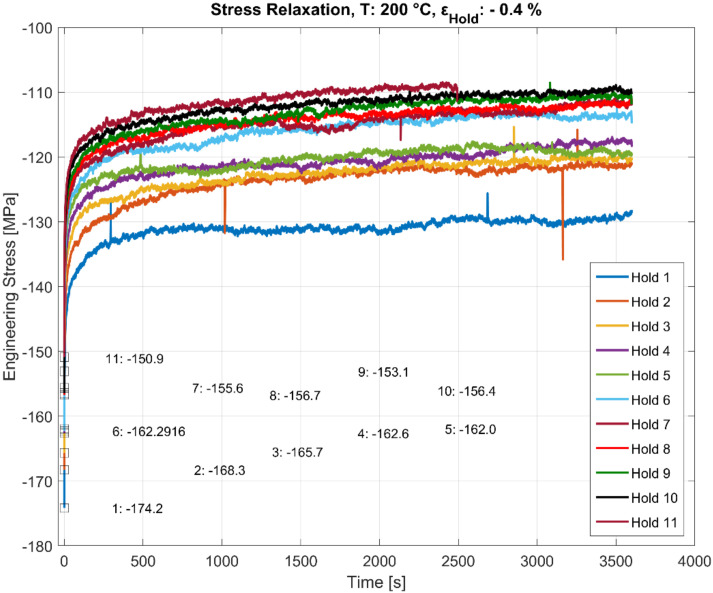
Effect of hold time on stress relaxation at 200 °C with alternating fatigue loading and hold period. The start (trough) values at the beginning of each hold period (numbered 1–11) are stated in MPa.

**Figure 12:**
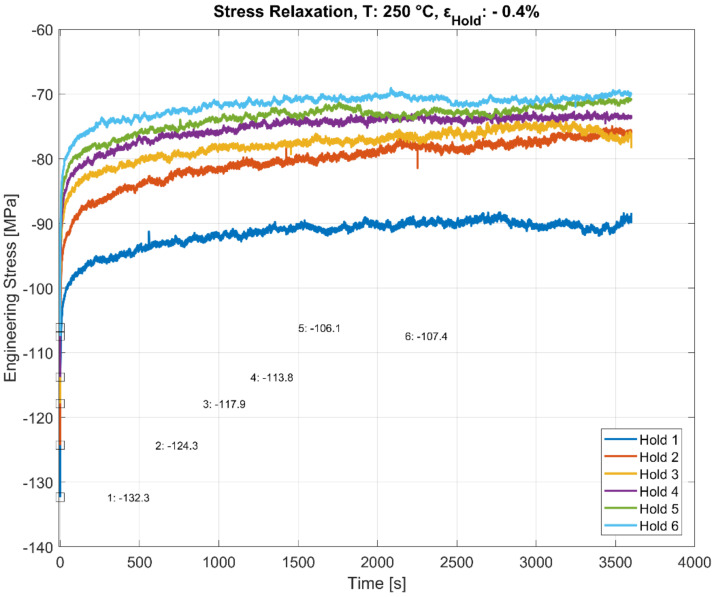
Effect of hold time on stress relaxation at 250 °C with alternating fatigue loading and hold period. The start (trough) values at the beginning of each hold period (numbered 1–6) are stated in MPa.

As illustrated in Figure 6, Figure 7, Figure 8 and Figure 9, the stress relaxation behaviour, with the saturation stress in particular, seem to be strongly dependent on the stress response during the initial rapid loading towards the hold strain and the temperature at which the loading occurs. The amount of stress relaxation, quantified by the difference between the stress at the beginning and the end of the dwell period, is often load sequence and material-dependent [14,27]. When combined with the fatigue loading in between the hold times, as illustrated in Figure 5, the initial stress response due to the rapid loading to the target compressive dwell strain serves as an indicator for the cyclic hardening/softening behaviour of the alloy when the dwell periods are interspersed with the cyclic loading, as illustrated in Figure 10, Figure 11 and Figure 12. The plots present the stress relaxation behaviour of the alloy when held at a compressive total strain of −0.4% for 3600 s at the three different temperatures. The square markers indicate the initial starting stresses after the rapid loading to the compressive strain value for each of the successive hold time sequence. The stress evolution for each of the successive hold time periods during the combined fatigue-dwell cycle, as illustrated in Figure 5, is indicated in Figure 10, Figure 11 and Figure 12 using the nomenclature Hold ‘x’, where ‘x’ indicates the hold sequence number incrementally in the order of ‘hold time’ execution. They are labelled at proportional heights depending on the initial starting stress; the minimum (trough) value. The plots indicate that the material softens with cyclic loading at each of the elevated temperature tests, namely, 150, 200, and 250 °C. Moreover, of interest is the differing stress level, but similar stress decay reached with prolonged hold times that are proportional to the initial starting stress at each of the temperatures tested. This indicates that the initial elastic/plastic contributions to the stress response from the rapid loading have a significant influence on the subsequent stress level, but not on the relaxation process.

### 3.4. Effect of Dwell Time on Peak and Trough Stress Evolution

**Figure 13:**
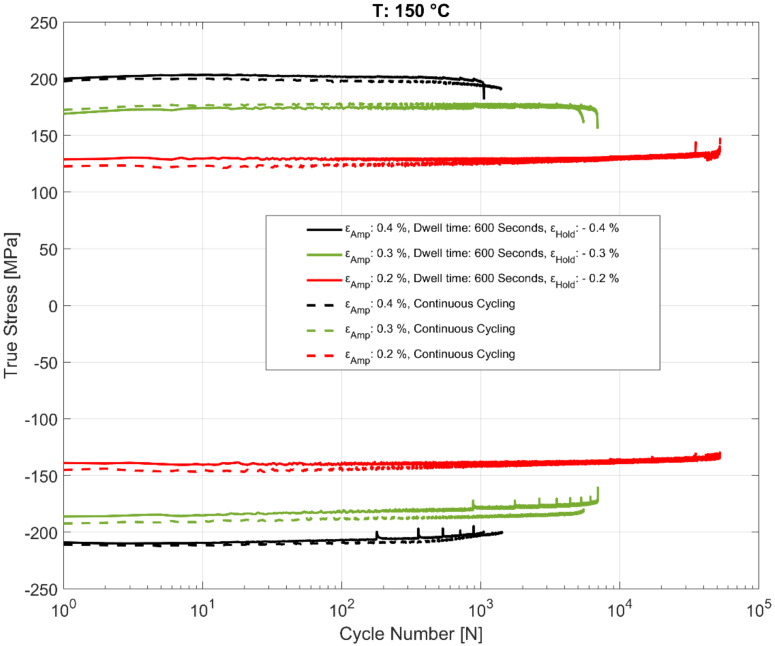
Evolution of peak and trough stress response in strain-controlled fatigue tests with and without hold time at 150 °C.

**Figure 14:**
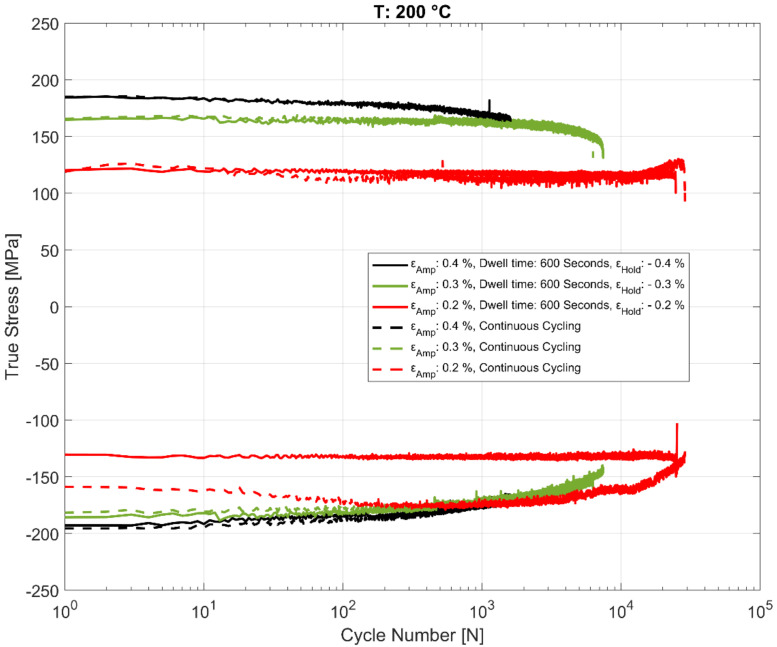
Evolution of peak and trough stress response in strain-controlled fatigue tests with and without hold time at 200 °C.

**Figure 15:**
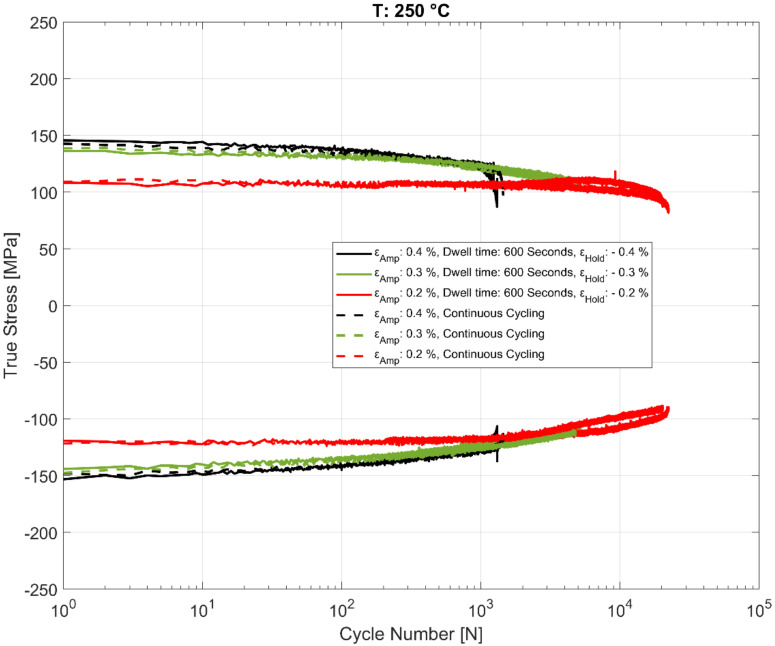
Evolution of peak and trough stress response in strain-controlled fatigue tests with and without hold time at 250 °C.

The effect of the hold time on the cyclic deformation and component life is of practical interest and has been studied in detail in this work. The evolution of the peak tensile and compressive stresses for the cyclic strain controlled tests with the recurrent hold time of 600 s at the various temperatures are summarized in Figure 13, Figure 14 and Figure 15 for various total strain amplitudes and temperatures. To enable comparison with strain controlled fatigue tests without any hold times, reference data from prior research work [24] were also added to the plots. As is evident, the strain-controlled tests while showing minor perturbations in the stress response after the compressive strain hold time tend to shake down within 2–3 cycles and seem to have very little effect on the subsequent cyclic hardening/softening behaviour of the alloy. As can be seen in the plots, no meaningful change in the life of the fatigue specimens is observed when compared with the continually loaded strain-controlled fatigue tests without any hold time in between at any of the tested temperatures or loads. A similar behaviour is observed for the tests with a hold time of 3600 s as well, implying that the fatigue behaviour of the alloy is not meaningfully affected by the dwell time at compressive strains as might be experienced by the hot sections of the material structure in the cylinder head of an internal combustion (IC) engine during the operation. Also of interest is the difference in the evolution of the peak compressive stress at 200 °C for the tests with ε_amp_ = 0.2% when comparing the continuously loaded and the strain controlled tests with the dwell time interspersed as shown in Figure 14. This can be attributed to the scatter often observed with cast aluminium structures, where the complex material microstructure from the differing cooling rates often results in a considerable scatter of the deformation and fatigue properties between test replicas [23,24].

### 3.5. Effect of Hold Times at Room Temperature

**Figure 16:**
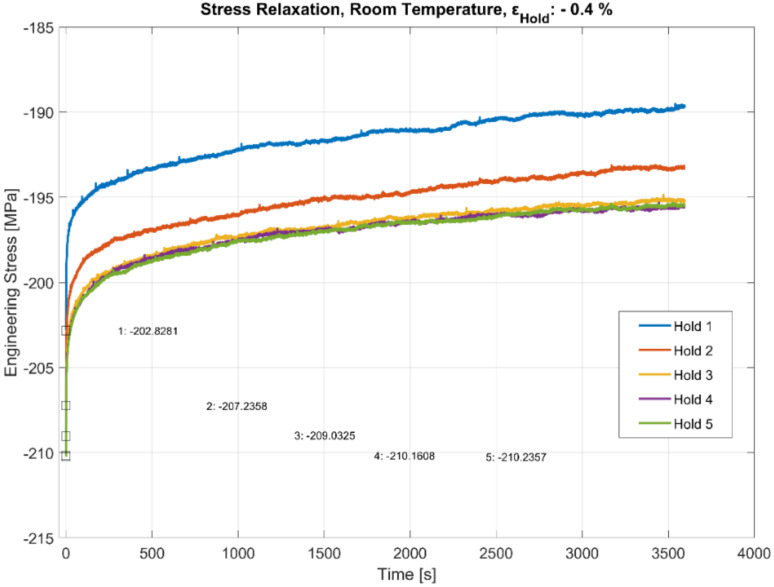
Effect of hold time on stress relaxation at room temperature with alternating fatigue loading and hold period. The start (trough) values at the beginning of each hold period (numbered 1–5) are stated in MPa.

While creep mechanisms at high temperatures are well documented [8,28,29], we find that the stress relaxation occurs even at room temperature in the A356-T7 + 0.5 wt.% Cu aluminium alloy when the material compressive strain is held constant after loading, as illustrated in Figure 16. While the mechanisms are different at different temperatures [29], it is essential to capture all the time dependent deformation behaviour in our rate-dependent constitutive models, especially if the dwell time has an impact on the fatigue behaviour of the material during thermo-mechanical fatigue loadings. Also evident in the room temperature tests presented in Figure 16 is the non-linear cyclic hardening behaviour at room temperature with the starting stresses before each hold time increasing with subsequent cycling during the test cycle. In this experiment, it seems this hardening saturates after some 25% of the number of cycles to failure.

## 4. Discussion of the Experimental Results

### 4.1. General

The samples for testing are extracted from the valve bridge area of the cylinder heads, which is particularly sensitive to the thermo-mechanical fatigue failures associated with the engine start–stop cycle [26]. The zone between the exhaust valves has been shown to be particularly vulnerable to the increased thermal loads, where the surface temperatures can often reach up to 300 °C depending on the engine cooling architecture [9]. The microstructure of the material in the cylinder head is often not uniform, and the dendritic arm spacing, in particular, tends to be more refined closer to the flame deck where the solidification rates are much higher than the rest of the structure [9,10]. To enable reliable development of numerical models, the samples for testing were extracted directly from the cylinder heads in order to, as closely as possible, mimic the material microstructure in the serially produced cylinder heads. This microstructure is often difficult to replicate with specially cast test bars [10,30,31]. Scatter in the observed properties is often expected on account of the variation of the microstructure within the cylinder head as well as the difference arising from the different moulds/batches of the production process [10]. The results and interpretations are affected by the choice to use material more relevant for the application than material from more well-defined laboratory castings. The natural spread in properties among the test results of this study need to be accounted for in the design.

### 4.2. Stress Relaxation during the Hold Time

#### 4.2.1. Stress Relaxation at Elevated Temperatures

As with most metals subjected to hold time during high temperature fatigue cycles [26], most of the stress relaxation occurs in the initial few minutes for the tested A356-T7 + 0.5 wt.% Cu alloy, where the relaxation is exponentially decaying. The stress relaxation rate experienced by the material during the hold time stabilizes fairly quickly when the material is held in compression. It is well established that the mechanisms responsible for creep in metallic materials are the same, contributing to stress relaxation [13,32,33] even if the resulting dislocation structures and their densities could be markedly different [34]. Prior research into stress relaxation in aluminium attributes the stress relaxation exhibited by the material primarily to dislocation creep in the initial stages, where we observe decreasing relaxation rates [13], and later on to diffusional creep and dislocation climb mechanisms [18,35]. The longer the hold time, the greater the conversion of the initial elastic strain to creep strain when the material is held at a constant total strain while the initial deformation is predominantly elastic and plastic [8,35,36].

#### 4.2.2. Stress Relaxation at Room Temperature

While the viscous behaviour at room temperature is often overlooked in modelling the cyclic plastic behaviour of aluminium alloys, we observe from our studies that the magnitude of stress relaxation at room temperature is just as significant compared with those at elevated temperatures. The stress relaxation at room temperature is mainly attributed to dislocation glide processes, which are often diffusion-independent. The creep at elevated temperatures and higher loads is also attributed to dislocation glide, but with thermal activation of the dislocation determining the stress loss rate [29]. Numerous studies have examined the room and medium (up to 0.4*T_m_*, where *T_m_* is the melting temperature) temperature creep behaviour of aluminium and its alloys in detail [37,38,39,40,41]. Ueda et al. [39] observed that the room temperature creep deformation of the aluminium alloy occurred at stresses higher than the 0.2% proof stress and that the grain size had no influence on the creep rate of the material at RT. They further identified the activation of secondary slip planes and their propagation to adjacent grains. They concluded that the creep behaviour in aluminium is caused by dislocation motion often with tangled dislocation structures and with diffusion having a negligible contribution [38,39]. Prasad et al. [41] also attribute the ease of cross-slip in face centred cubic (FCC) metals owing to the increased number of slip systems aiding the low temperature creep behaviour of aluminium alloys. This compressive strain dwell and the associated stress relaxation at room temperature does not, however, affect the number of cycles to failure of the material as with most engineering metals [42].

### 4.3. Effect of Hold Time on Number of Cycles to Failure

Numerous studies have shown the fatigue resistance reducing the effects of hold time periods for different materials exposed to high temperatures and harsh working environments [43,44,45,46]. While fatigue damage during dwell-fatigue testing is often only associated with tensile loadings during the hold period [14,47], it is not uncommon for materials to exhibit a reduced number of cycles to failure even with hold periods in compressive loading [20]. Except for fatigue, an important factor in the longevity of the material subjected to thermo-mechanical load cycles with dwell times of strain comes from the microstructural changes when the phases present initially are unstable at elevated temperatures [26], or the operating environment causing the materials to undergo time-dependent damage processes like embrittlement through oxidation, chlorination, and so on, which could potentially reduce the subsequent load carrying capacity of the structure [17,26,29,45].

**Figure 17:**
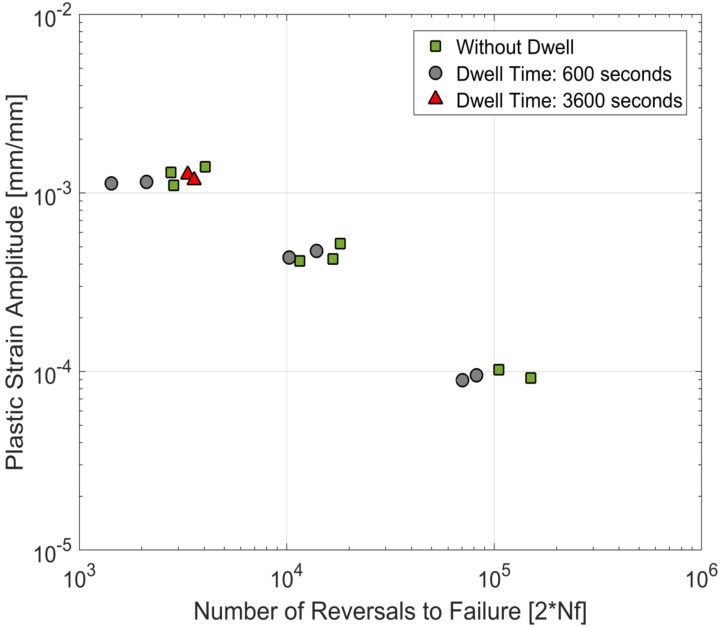
Strain life curves at 150 °C—comparison of fatigue tests with and without compressive dwell times.

**Figure 18:**
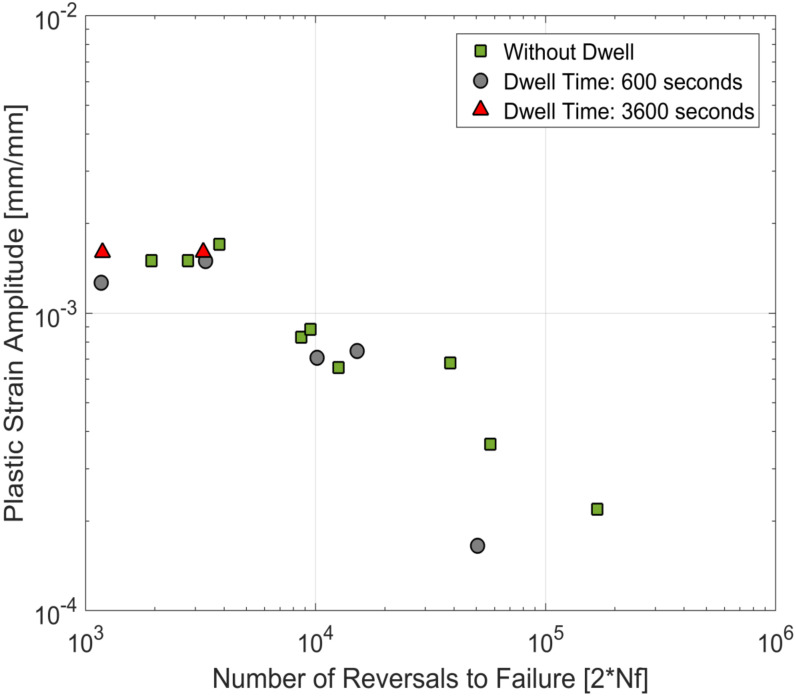
Strain life curves at 200 °C—comparison of fatigue tests with and without compressive dwell times.

**Figure 19:**
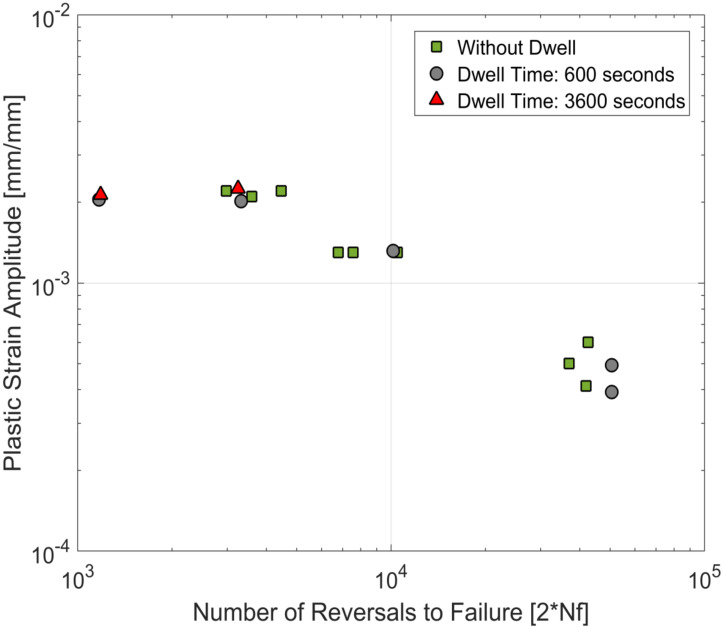
Strain life curves at 250 °C—comparison of fatigue tests with and without compressive dwell times.

The life curves obtained from the tests in this study are plotted together with the life curves with continuous loadings of the same material from prior research [23,24] and are presented in Figure 17, Figure 18 and Figure 19. For the tested A356-T7 + 0.5 wt.% Cu cast aluminium alloy, while the scatter in the measured lives (as is usually observed in most cast aluminium alloys) makes it challenging to conclude about the effect of dwell times on the number of cycles to failure of the material, the tests with the hold times of 3600 s help paint a clearer picture, as no significant reduction of fatigue lives is observed even for the tests at 250 °C. For the tested dwell times, it is reasonable to conclude that no significant environmental or microstructural damage is observed for the tested alloy.

## 5. Numerical Modelling

For a cost-efficient engine development program, material models that are included in commercial finite element codes are preferred. Furthermore, as engineers, we must balance between the required level of detail and the computational cost of more complex models. Consequently, we will neglect the material hardening/softening owing to ageing of the alloy. Additionally, complex models with many parameters are difficult to calibrate and pose challenges when it comes to reproducibility according to Tramper et al. [7]. For the same reasons, they also advocate decoupled constitutive and life models.

As previously shown, the dwell times during compression seem to have little impact on the tested material’s cyclic hardening behaviour and its durability. These results indicate that a rate-independent model can be applied. However, in the real application, low-amplitude high cycle fatigue loading occurs in the cylinder head during the dwell time. This loading is associated with the high frequency combustion cycles in contrast to the high amplitude, low frequency thermo-mechanical low cycle fatigue loading associated with the engine start–run–stop cycles. Beck et al. [19] observed that the stress relaxation during the dwell may cause combustion loading to generate tensile stresses in the structure. These tensile stresses need to be predicted accurately by the constitutive model as they cause a reduction in the number of cycles to failure. Hence, a rate-dependent model should be employed to ensure an accurate life prediction.

The Chaboche model is suitable for cyclic loading as it combines exponentially saturating isotropic hardening with Armstrong–Frederick kinematic hardening. In the commercial finite element code Abaqus [48], it can be combined with the Cowper–Symonds overstress power law. This combined model can describe the rate-dependent cyclic plasticity, and a brief description is given below. The parameters for this phenomenological model are calibrated at discrete temperatures. These parameters are interpolated to simulate thermo-mechanical response of the alloy at intermediate temperatures.

Yield surface:

(1) $$F=f\left(\sigma -\alpha \right)-\sigma^{0}=0$$

where $f\left(\sigma -\alpha \right)$ is the equivalent Mises stress with respect to the back stress α and $\sigma^{0}$ is the yield stress. The stress tensor σ is calculated as follows:

(2) $$\sigma =E:\varepsilon_{e}$$

where E is the fourth-order isotropic elastic stiffness tensor and the total strain **ε** is defined as follows:

(3) $$\varepsilon =\varepsilon_{e}+\varepsilon_{p}$$

The associated plastic flow rule is defined as follows:

(4) $${\dot{\varepsilon }}^{p}={\dot{\bar{\varepsilon }}}^{p}\frac{\partial F}{\partial \sigma }$$

where ${\dot{\varepsilon }}^{p}$ is the rate of the effective plastic strain.

Isotropic hardening: exponential law:

(5) $$\sigma^{0}=\sigma {¦}_{0}+Q_{\infty }\left(1-e^{-b{\bar{\varepsilon }}^{pl}}\right)$$

Here, $\sigma {¦}_{0}$ is the yield at zero plastic strain and $Q_{\infty }$ is the maximum change in the size of the yield surface. The parameter b describes the rate of change of the size of the yield surface with accumulated plastic strain.

Non-linear kinematic hardening: Armstrong–Frederick model:

(6) $${\dot{\alpha }}_{k}=C_{k}\frac{1}{\sigma^{0}}\left(\sigma -\alpha \right){\dot{\bar{\varepsilon }}}^{pl}-\gamma_{k}\alpha_{k}{\dot{\bar{\varepsilon }}}^{pl}$$

with the overall backstress being an additive partition described by $\alpha =\sum_{k=1}^{N}\alpha_{k}$. $C_{k}$ are the initial kinematic hardening moduli and the rate at which the initial kinematic hardening moduli decreases with accumulated plastic deformation is determined by the parameter $\gamma_{k}$.

Overstress power law: Cowper–Symonds model:

(7) $${\dot{\bar{\varepsilon }}}^{pl}=D\;{\left(R-1\right)}^{n}\;\mathrm{for}\;\bar{\sigma }\geq \sigma^{0}$$

where $\bar{\sigma }$ is the rate dependent yield stress. R defines the ratio of the yield stress at a specified strain rate to the static yield strength $\sigma^{0}$. So,

(8) $$\bar{\sigma }=f\left(\sigma -\alpha \right)$$

(9) $$R=\frac{\bar{\sigma }}{\sigma^{0}}=\frac{f\left(\sigma -\alpha \right)}{\sigma^{0}}$$

The material model parameters are the multiplier D and the exponent n. A detailed explanation of the implementation of the models can be found in [48].

### 5.1. Calibration Procedure and Results

The stress response during the first set of cycles and the first dwell time is used to calibrate the model parameters. The stress–strain data of the cyclic loads with ε_amp_: 0.4%, R_ε_ = −1, and the subsequent hold for 600 s at ε_tot_ = −0.4% are used for the calibration of the test temperatures 150, 200, and 250 °C. For the room temperature model, the dataset with the hold time of 3600 s is used instead. The parameter optimization is carried out using the open source ‘matmodfit’ program [49] with its implementation as described in the research work by Meyer et al. [50]. We use the Nelder–Mead gradient-free optimization algorithm [51] to minimize the square stress difference between the simulations and the experiments. The optimized parameter values are presented in Table 4. One linear and one non-linear kinematic backstress are used to capture the translation of the yield surface with plastic deformation. The linear part of the kinematic hardening model is obtained by constraining the parameter $\gamma_{2}$ to zero. The stiffness of the material is calculated from the initial tensile loading for each of the temperatures and the evolution of the yield surface is estimated from the continuous cyclic loading data. The kinematic hardening moduli, $C_{1}$ and $C_{2},$ and the rate dependent multiplier D decrease with temperature, whereas the exponent n increases.

The parameters that do not show monotonic variation are the two saturation rate controlling parameters: the kinematic saturation rate controlling parameter $\gamma_{1}$ and the isotropic saturation rate controlling parameter b. However, given the small increments of temperature of 50 °C at temperatures above 150 °C when significant changes in the plastic deformation of the material are observed [23,24], a stable interpolation of the deformation behaviour can be obtained at temperatures in between the modelled discrete test temperatures for thermo-mechanical loading simulations.

**Figure 20:**
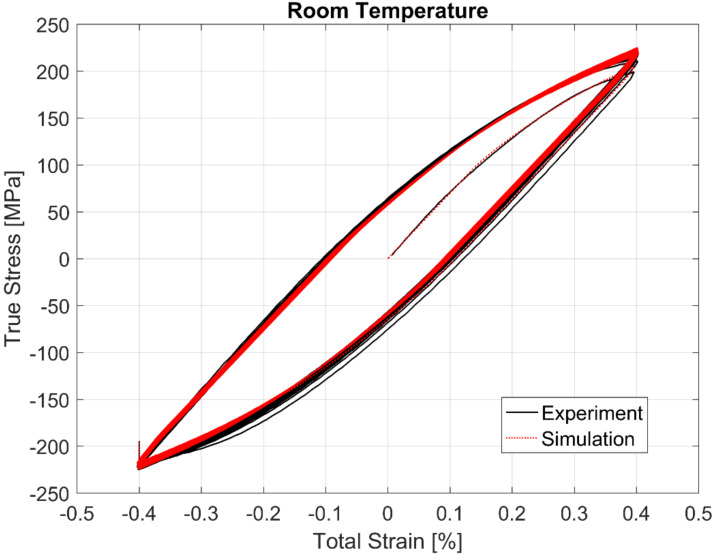
Experimental vs. visco-plastic model prediction at room temperature (RT).

**Figure 21:**
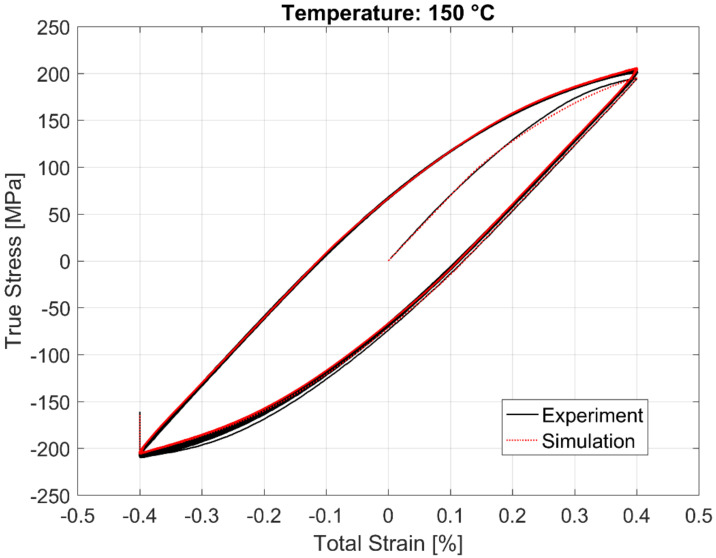
Experimental vs. visco-plastic model prediction at 150 °C.

**Figure 22:**
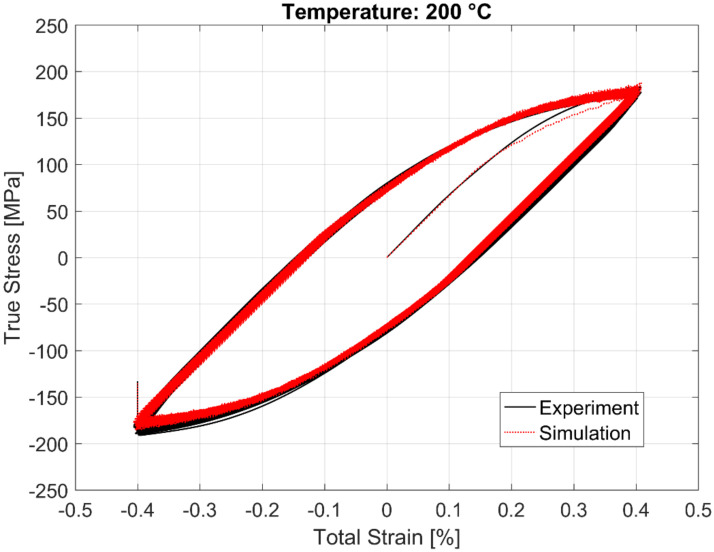
Experimental vs. visco-plastic model prediction at 200 °C.

**Figure 23:**
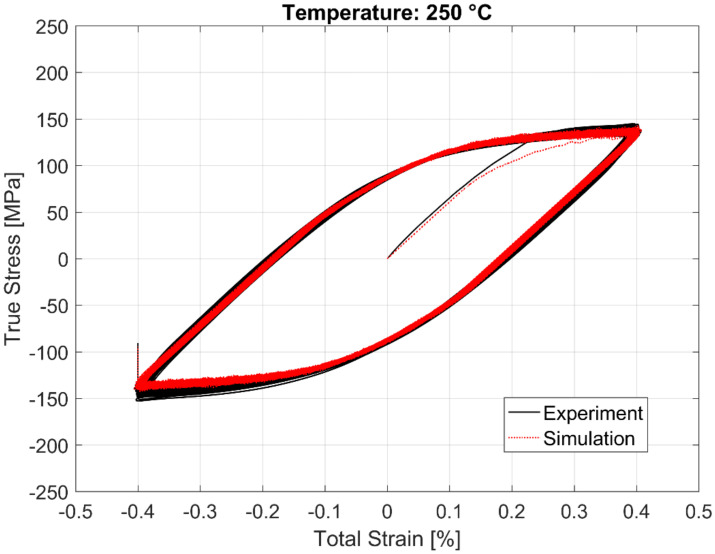
Experimental vs. visco-plastic model prediction at 250 °C.

The calibrated model prediction results from a single-point simulation and the experimental data are presented in Figure 20, Figure 21, Figure 22 and Figure 23.

**Figure 24:**
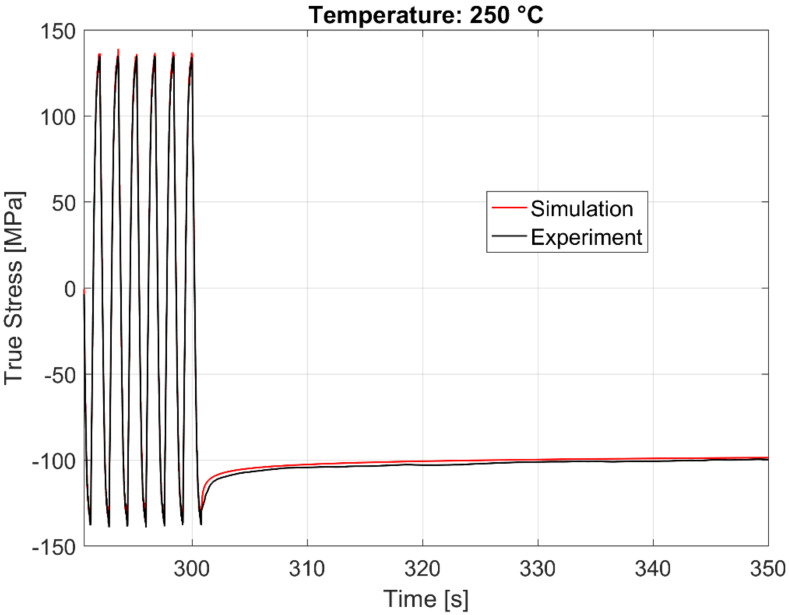
Time history of the experimental vs. visco-plastic model prediction at 250 °C.

**Figure 25:**
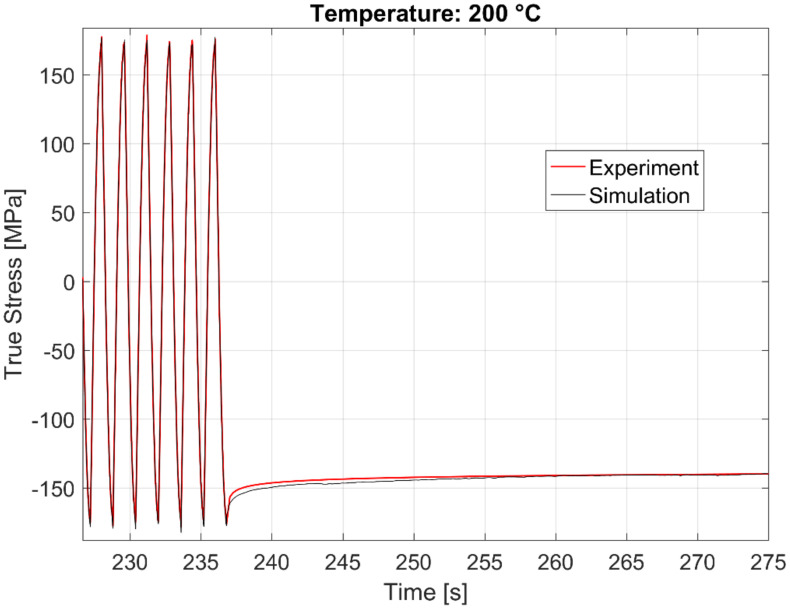
Time history of the experimental vs. visco-plastic model prediction at 200 °C.

**Figure 26:**
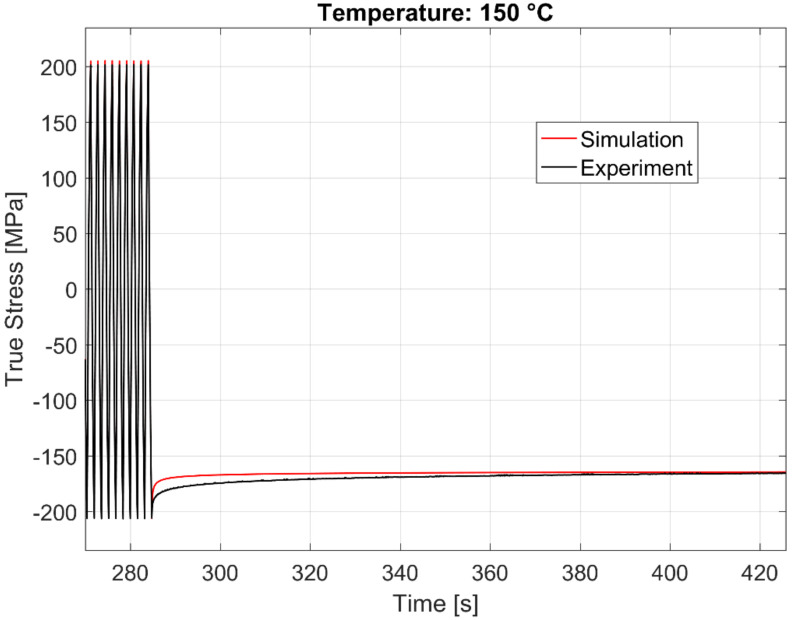
Time history of the experimental vs. visco-plastic model prediction at 150 °C.

The time history of the model prediction of the cyclic continuum stress and the dwell time stress relaxation at 250, 200, and 150 °C just before and after the dwell commences is presented in Figure 24, Figure 25 and Figure 26 respectively.

### 5.2. Verification of the Model

**Figure 27:**
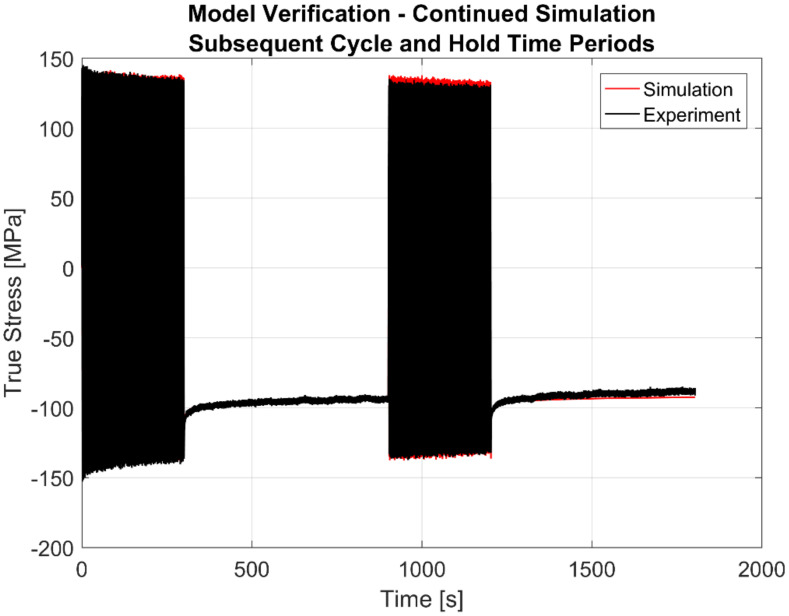
Simulation of continued loading of the second cycle and hold times for verification at 250 °C.

**Figure 28:**
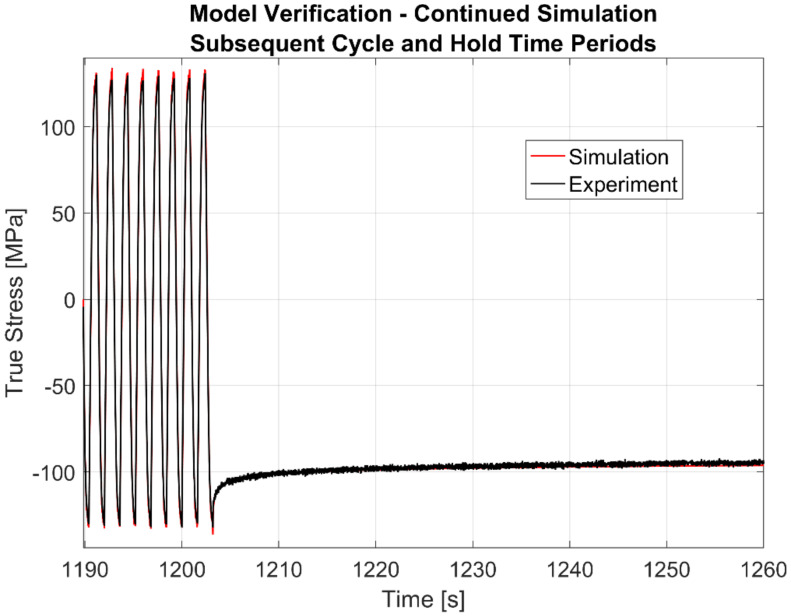
Simulation of continued loading of the second cycle and hold times for verification just before and after the second dwell commences at 250 °C.

To show the predictive capability of the model, the simulation is carried out with the second loading sequence of the strain cycling–dwell tests. The result for a sample simulation at 250 °C is shown in Figure 27. A magnified image showing a few load cycles prior to the second dwell time period followed by the initial 50 s of the dwell period itself is further shown in Figure 28.

### 5.3. Analysis of the Modelling Results and Limitations of the Model

While the cyclic deformation and the stress relaxation associated with the dwell time period of the tested A356-T7 + 0.5 wt.% Cu alloy can be modelled to a sufficient degree of accuracy using a combination of the Chaboche and the rate-dependent Cowper–Symonds overstress power law models, as demonstrated above, the calibrated model exhibits its limitations in accurately predicting the cyclic deformation behaviour of the initial few cycles (cycles 1−3), especially at elevated temperatures. By increasing the emphasis on the initial cycles in the calibration procedure, these can be captured more accurately. However, such a modification reduces the modelling accuracy of the remaining cycles. The present approach is thus motivated by the initial cycles’ negligible influence on the life predictions. The model prediction also deviates slightly from the initial decreasing creep/relaxation rate from the experimental data just after the dwell time begins. However, in the application, the durability of the material is more severely affected by the stress state after the initial relaxation state owing to the superimposed high frequency load cycles associated with the combustion process [19]. Hence, the prediction of the calibrated model of the stress state during this later stage of the dwell time period plays a more significant role in the CAE determination of the durability of the structure than the initial decreasing creep rate, and hence has been given higher priority during the calibration. While it is possible to get a better agreement through all stages of the dwell-fatigue loading cycles using additional material parameters, the tradeoff is often the computational costs of the CAE simulations, which is further exacerbated while simulating complex structures like cylinder heads.

In summary, the stress-strain, load-response history of the alloy can be obtained with the above mentioned limitations using the presented visco-plastic models for various loads that cylinder heads and other such structures made of the studied A356-T7 + 0.5 wt.% Cu cast aluminium alloys are subjected to with operational transient thermomechanical loads. The obtained load-response history can subsequently be combined with suitable life models to get an estimation of the durability of the components and help with CAE design iterations.

## 6. Conclusions

A356-T7 + 0.5 wt.% Cu cast aluminium alloys extracted from specially cast engine cylinder heads are subjected to cyclic strain-controlled fatigue tests with interspersed dwell times at compressive strains at various temperatures and load levels. Dwell times of 600 s and 3600 s were used to study the effect of the length of the dwell times on the fatigue lives.

- The material exhibits a significant stress relaxation at all temperatures and load levels with a rapidly decreasing stress relaxation rate.
- The magnitude of stress relaxation is influenced significantly by the load level. This effect is stronger at the lower test temperatures of 150 °C than at the higher test temperature of 250 °C. This can be attributed to the plastic hardening behaviour of the alloy at lower temperatures, while the material owing to excessive stress relaxation shows insignificant hardening at 250 °C.
- The dwell times at constant compressive strains have no discernible influence on the cyclic hardening behaviour or the fatigue life of the material, even at elevated temperatures.
- The visco-plastic deformation behaviour can be modelled with a high degree of accuracy using a combination of the Chaboche combined non-linear kinematic and isotropic mixed hardening model and the rate-dependent Cowper–Symonds overstress power law model.
- Further research using TMF testing is strongly recommended along the lines of the work by Beck et al. [19]. A detailed study of the combined effect of stress relaxation associated with the low frequency thermal start–stop cycle and superimposed high frequency loads associated with the combustion cycles is needed to get a more complete picture of the effect of these loads on the durability of cylinder heads.

## Figures and Tables

**Table 1:** Chemical composition of the A356-T7 + 0.5 wt.% Cu alloy in wt.%.

Si	Cu	Mg	Ti	Fe	Mn	B	Others	Al
6.8	0.53	0.35	0.12	0.10	0.07	0.0012	<0.05	Bal

**Table 2:** Summary of the test plan. RT, room temperature.

Test Series	Test Temperatures	Load Levels	Replicas
Series A: Strain controlled tests with a hold time of 600 s	150, 200, and 250 °C	Total Strain Amplitudes ε_TotAmp_: 0.2%, 0.3%, and 0.4% Strain Ratio: R_ε_ = −1	2 at each load-temperature combination
Series B: Strain controlled tests with a hold time of 3600 s	RT, 150, 200, and 250 °C	Total Strain Amplitude ε_TotAmp_: 0.4% Strain Ratio: R_ε_ = −1	2 at each temperature

**Table 3:** Extensometers used.

Extensometer	Temperature
Instron 2620-603 axial clip-on dynamic extensometer (Instron, Norwood, MA, USA)	RT & 150 °C
Epsilon 3555-010M-020 high temperature 146 axial capacitive extensometer (Epsilon Technology Corporation, Jackson, WY, USA).	200 & 250 °C

**Table 4:** Visco-plastic model parameters of the Chaboche + Cowper–Symonds rate-dependent model.

Temperature °C	Young’s Modulus [GPa]	Yield $\sigma {¦}_{0}$ [Mpa]	Kinematic Hardening Parameter $C_{1}$ [Mpa]	$\gamma_{1}$ [-]	Kinematic Hardening Parameter $C_{2}$ [MPa]	$\gamma_{2}$ [-]	$Q_{\infty }$ [MPa]	**Hardening Parameter**b [-]	**Multiplier** D [-]	**Exponent** n [-]
RT	69.85	90.00	156,178	3266	41,437	0	17	0.6141	0.0179	1.4422
150	69.55	64.65	154,190	2519	22,216	0	−1.5	0.4601	0.0118	3.8595
200	66.33	54.50	115,350	1869	7286	0	−10	0.6161	0.0062	6.9943
250	60.97	41.79	80,620	2161	3492	0	−40	0.0315	0.0018	6.9943
